# Is the Small Ossicle of Type 1 Accessory Navicular a Cause of Foot Pain?—A Case Report

**DOI:** 10.3390/medicina59091562

**Published:** 2023-08-28

**Authors:** Takuji Yokoe, Kazushi Uemichi, Takuya Tajima, Etsuo Chosa

**Affiliations:** 1 Division of Orthopaedic Surgery, Department of Medicine of Sensory and Motor Organs, Faculty of Medicine, University of Miyazaki, 5200 Kihara, Kiyotake, Miyazaki 889-1692, Japan; 2 Department of Orthopaedic Surgery, Kobayashi City Hospital, 2235-3 Hosono, Kobayashi, Miyazaki 886-0004, Japan

**Keywords:** accessory navicular, classification, small ossicle, surgery

## Abstract

*Background and objectives*: An accessory navicular (AN) bone is often classified into types 1–3 according to the Veitch classification, and symptomatic type 1 patients usually receive non-surgical treatment. However, there are cases in which AN cannot be classified into one of these three types using this classification system, and the small ossicle of type 1 AN may not be the cause of foot pain. This report aimed to present a case of symptomatic type 1 AN that required surgical treatment without the excision of the small ossicle after long-term conservative treatment had failed. *Case presentation:* A 15-year-old girl who was diagnosed with symptomatic type 1 AN was referred to our department. Medial-side foot pain had prevented her from playing soccer well. She had been treated conservatively for type 1 AN for more than 12 months at several orthopedic clinics. Tenderness of the prominent navicular tubercle was identified, and computed tomography and magnetic resonance imaging findings suggested that the cause of her foot pain was derived from the prominent navicular tubercle not the small ossicle itself. Osteotomy of the prominent navicular tubercle with the advancement of the tibialis posterior tendon, without excision of the ossicle, was performed. At the 12-month follow-up examination, she was completely free from foot pain, and the patient-reported outcome measures were excellent. She now plays soccer at the pre-injury level. *Conclusions:* We report the case of a patient with symptomatic type 1 AN who underwent osteotomy of the prominent navicular tubercle with advancement of the tibialis posterior tendon, without excision of the ossicle, and who showed favorable short-term clinical outcomes. The evaluation of symptomatic patients with AN based on the Veitch classification alone may lead to inappropriate management. The small ossicle of type 1 AN was not the cause of foot pain in the present case.

## 1. Introduction

An accessory navicular (AN) bone is one of the most common foot accessory ossicles, with a prevalence of between 4–21% [1,2]. This ossicle is also referred to as os naviculare secundarium, os tibiale externum, and os naviculare accessorium. The AN is generally located in the posteromedial part of the foot, proximal to the navicular bone, and is embedded in the tibialis posterior tendon (TPT). A large proportion of patients with AN are asymptomatic. However, conservative treatment including shoe-wear modification, shoe inserts, non-steroidal anti-inflammatory medication, and ankle orthosis is needed for symptomatic patients [2,3]. When non-surgical treatment fails, surgery is generally recommended to get rid of symptoms. To date, it remains unclear why some patients suffer from symptoms of ANs while others do not. Clinically, the classification system proposed by Veitch is frequently used [4]: type 1, the ossicle is independent of the navicular bone; type 2, the ossicle is connected to the navicular bone through cartilaginous or fibrocartilaginous tissue; and type 3, the ossicle is united to the navicular tuberosity through a bony bridge. Patients with type 2 AN often become symptomatic and will require surgical treatment when conservative treatment fails [5,6,7,8,9]. A simple excision or a Kidner procedure (excision of the ossicle with TPT advancement) is a gold standard surgical procedure. Although fewer clinical studies have been reported concerning the surgical treatment of type 3 AN compared to that of type 2 AN, simple excision or the Kidner procedure have also been considered when conservative treatment fails [3,7]. Symptomatic patients with type 1 AN are often treated non-surgically, and few studies have reported on its surgical treatment [7]. However, is the small ossicle of type 1 AN really the cause of a patient’s symptoms (pain)? Coughlin et al. classified ANs into 12 subtypes [10]. Additionally, multiple ANs have been reported by some authors [10,11,12]. Perdikakis et al. first reported the incidence of multiple ANs [11]. Of the 34 cases with ANs, 5 patients (14.7%) had multiple ANs. These studies suggested that the AN may demonstrate various morphologies. Therefore, it may not be appropriate to simply classify all ANs into three types according to the Veitch classification and treat them based on this classification. Considering the variety in the shapes of ANs, clinicians need to carefully perform physical examinations and interpret the imaging findings.

Herein, we report a case of symptomatic type 1 AN (according to the Veitch classification) that required surgical treatment without excision of the small ossicle itself after long-term (>12 months) conservative treatment had failed. The patient showed short-term favorable outcomes. Informed consent was obtained from the patient and her parents for the publication of this report and any accompanying images.

## 2. Case Presentation

**Figure 1:**
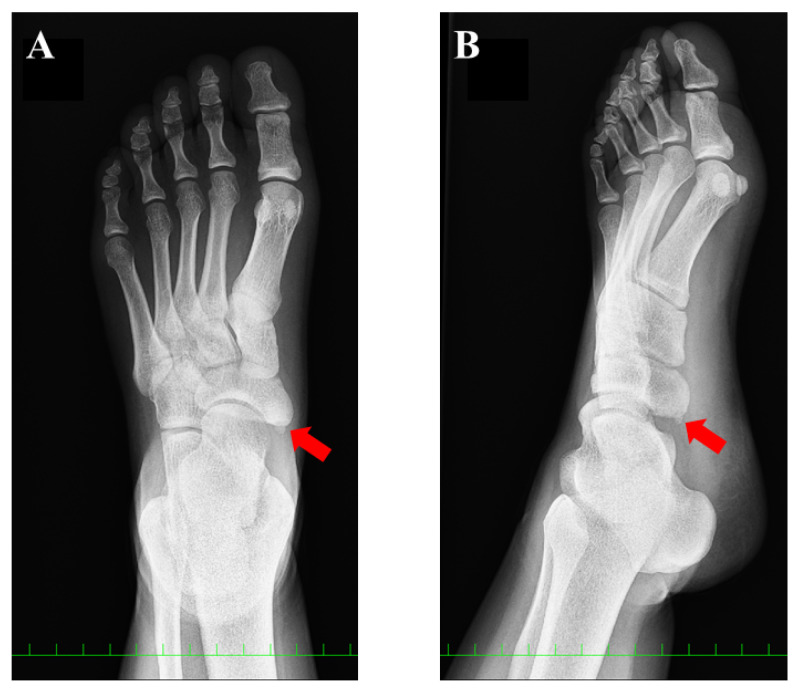
Preoperative plain radiographic findings: (**A**) anteroposterior view and (**B**) oblique view. The red arrow indicates the accessory navicular (AN). The AN was classified as type 1, according to the Veitch classification.

A 15-year-old girl was referred to our department for the assessment of left foot pain. She was diagnosed with AN, which was radiographically classified as Veitch type Ⅰ at a private orthopedic clinic (Figure 1).

**Figure 2:**
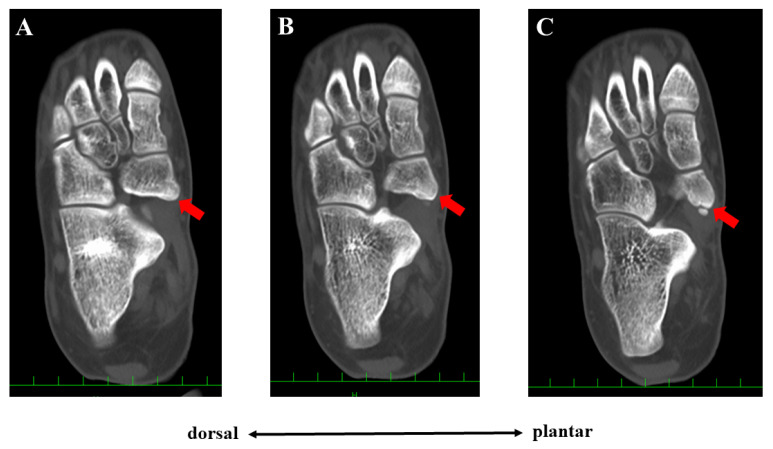
Preoperative computed tomographic findings. Three continuous axial scans at the lesion level are shown (**A**–**C**). The united ossicle of the accessory navicular (**red arrow**) showed a higher Hounsfield unit than the adjacent normal cancellous bone.

**Figure 3:**
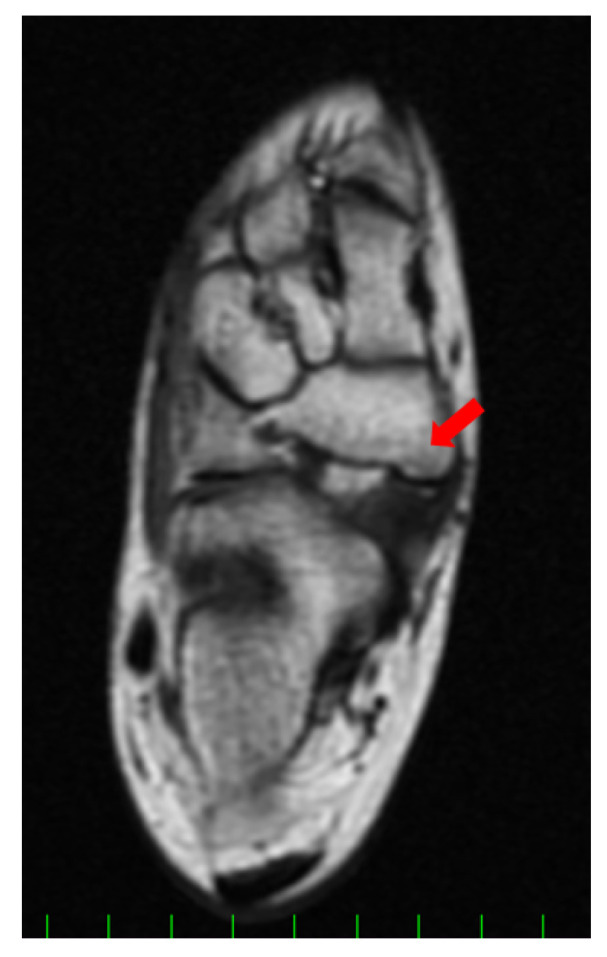
Preoperative magnetic resonance imaging (MRI) finding (axial image, T1WI). A low signal lesion was detected in the prominent part of the navicular (**red arrow**).

Conservative treatment including physiotherapy, medication for pain, and the application of an insole had been performed for more than one year but failed to completely remove her symptoms. She had been active in soccer, and her training experience was three years at the time of first presentation. She had tenderness at the navicular tuberosity with the exacerbation of pain while weight bearing. Plain radiographs did not show specific findings of flatfoot (Meary’s angle, 19.8 degrees; calcaneal pitch angle, 4.6 degrees) or hindfoot malalignment. Computed tomography (CT) showed a small AN and prominent navicular tuberosity with a high Hounsfield unit (HU) value (Figure 2). A low signal lesion was identified in the prominent part of the navicular bone on the T1-weighted image of the magnetic resonance imaging (Figure 3).

Magnetic resonance imaging (MRI) showed that the lesion was iso-signal in both the T1- and T2-weighted images. The preoperative American Orthopaedic Foot and Ankle Society (AOFAS) hindfoot–ankle score [13] and visual analog scale (VAS) were 67/100 and 4/10, respectively. The preoperative foot and ankle outcome score (FAOS) [14] was as follows: symptoms, 71.4/100; pain, 61.1/100; function, daily living, 100/100; function, sports and recreational activities, 30/100; and quality of life, 18.8/100.

**Figure 4:**
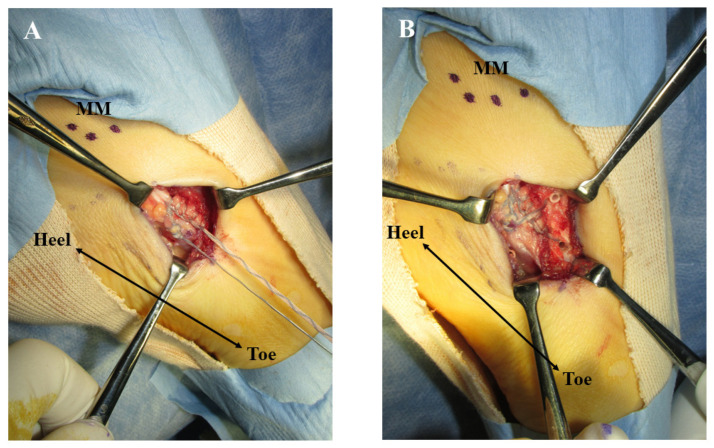
Surgical photographs. (**A**) After osteotomy of the prominent navicular and reduction of the volume of the attachment site of the tibialis posterior tendon (TPT), the Krackow technique was applied to the TPT using suture tape. (**B**) The TPT was stabilized to the navicular body with two knotless suture anchors in a suture bridge fashion. MM, medial malleolus.

**Figure 5:**
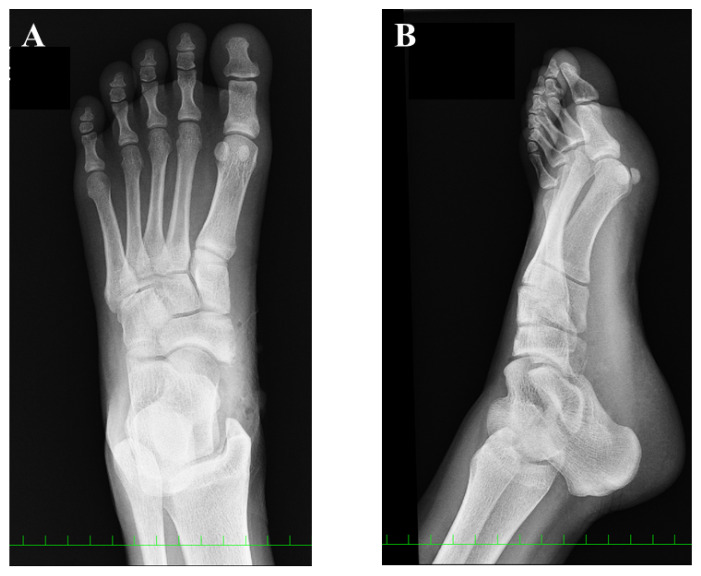
Postoperative plain radiographic findings: (**A**) anteroposterior view and (**B**) oblique view.

Surgery was performed with the patient in the supine position on the operating table under spinal anesthesia. A thigh tourniquet was applied at 270 mmHg. A longitudinal incision of about 30 mm was made over the tip of the navicular tuberosity, and the insertion of the TPT was exposed. The tendon sheath of the TPT was incised, and the course of the TPT was inspected. Findings of synovitis or degeneration of the TPT were not detected. The small ossicle itself was not removed considering that excision of the ossicle is associated with a risk of damaging the TPT. The prominent part of the navicular tuberosity was cut down using a small bone chisel while preserving the integrity of the TPT insertion. The osteotomized bone surface of the navicular was smoothened using a bone raspatory. Without disrupting the integrity of the TPT insertion, the volume of cancellous bone of the fragment was reduced and reshaping was performed. The Krackow technique was applied to the substance of the TPT using suture tape (FiberTape, Arthrex, North Naples, FL), and the TPT was stabilized to the navicular body using two knotless suture anchors (FiberTak^TM^, Arthrex, North Naples, FL, USA) in a “suture-bridge” fashion (Figure 4). After lavage with saline, the surgical wound was closed in a standard fashion. Postoperative radiographs are shown in Figure 5.

Postoperatively, the ankle was immobilized with a short plastic splint for two weeks, with no weight bearing for four weeks. Ankle range of motion exercises were initiated after two weeks postoperatively, and full range of motion (ROM) was allowed after four weeks following surgery. Weight bearing was gradually increased under ankle orthosis, and full weight bearing was allowed after eight weeks postoperatively. At four months after surgery, return to sports was allowed.

**Figure 6:**
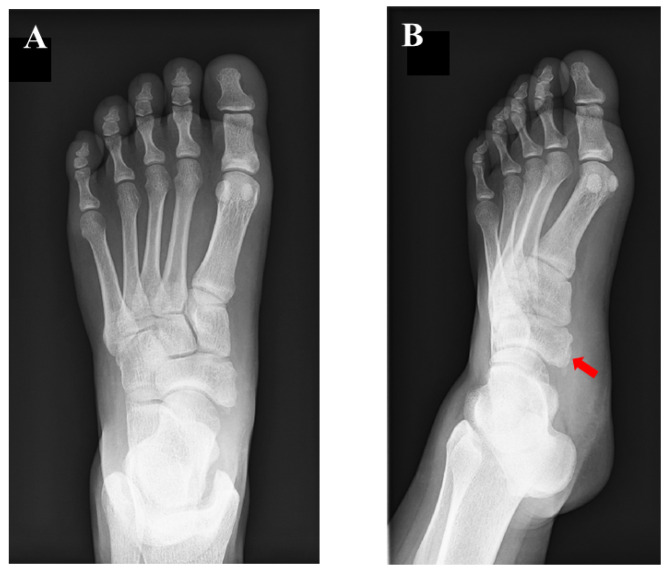
Plain radiographic findings at 12 months after surgery: (**A**) anteroposterior view and (**B**) oblique view. Union of the bony fragment with the tibialis posterior tendon to the navicular body was confirmed (**red arrow**).

**Figure 7:**
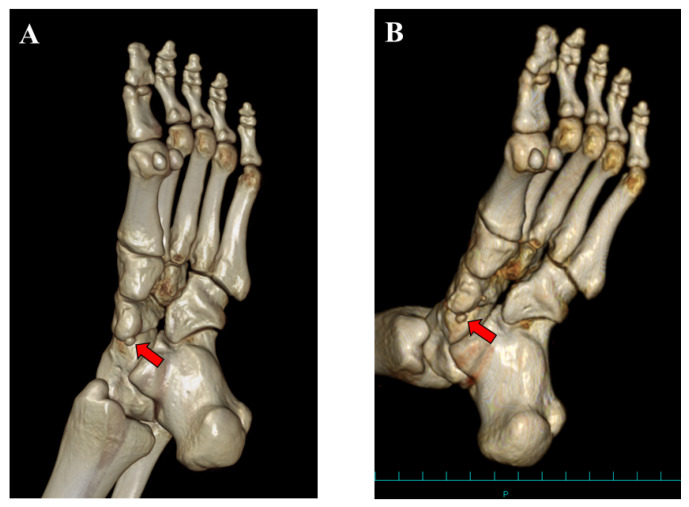
Three-dimensional computed tomographic (CT) findings: (**A**) preoperative and (**B**) postoperative CT findings at 12 months after surgery. A small ossicle (accessory navicular, **red arrow**) was present postoperatively.

At the 12-month follow-up examination, the AOFAS score and VAS were 100/100 and 0/10, respectively. The FAOS was as follows: symptoms, 100/100; pain, 100/100; function, daily living, 100/100; function, sports and recreational activities, 95/100; and quality of life, 100/100. Plain radiographs showed union of the bony fragment with the TPT to the navicular body (Figure 6). The CT findings showed the presence of the navicular bone (Figure 7). The patient has played soccer at the same pre-injury level without complaints.

## 3. Discussion

We present a case with symptomatic AN that had been treated conservatively for a prolonged time in accordance with the typical approach for Veitch type 1 AN, in which surgical intervention was subsequently needed to resolve the symptoms. In addition, in this case, the excision of the small ossicle was not needed to resolve the patient’s symptoms although osteotomy of the prominent navicular tuberosity and advancement of the TPT were performed.

The Veitch classification is the system most commonly used to evaluate patients with AN [5,6,7,8]. This classification is easy to understand and broadly used in clinical practice. However, various kinds of morphologies of the AN have been reported [2,10]. Furthermore, Perdikakis et al. first reported a case of multiple ANs [11]. Kalbouneh et al. reported that multiple ossicles were radiographically detected in 1.2% of cases with ANs [10]. The authors also reported that the larger ossicle was united to the navicular while the other round ossicle was clearly separated from the navicular bone, which was similar to the present case. In the present case, the patient had been treated conservatively for Veitch type 1 AN probably because few studies have reported the surgical management of type 1 AN [7]. However, tenderness was present at the united ossicle (navicular tuberosity), not at the separated small ossicle, indicating that the cause of foot pain was derived from the united ossicle. The lesion with high HU on CT images also suggested continued stress loading on the united ossicle. Therefore, from the viewpoint of the pathology, the present case could be classified as Veitch type 3 AN. Clinicians should not simply classify patients with AN into three types using the Veitch classification because this may lead to inappropriate management as presented in the present case.

Jegal et al. reported that conservative treatment for symptomatic AN was less effective for athletes than non-athletes (6.9% vs. 34%, *p* < 0.001) [15]. Some authors found that female patients were more likely to become symptomatic and require surgical procedures [3,16]. It was reported that female patients comprised 65% of simple excision and 73% of the Kidner procedure [3]. Accordingly, the activity level of the patient or their gender may affect the clinical course of patients with symptomatic AN. Wynn et al. reported the efficacy of conservative treatment for patients with symptomatic ANs [8]. Among 226 symptomatic ANs of 169 patients, 28% of the patients finally experienced complete pain relief without surgery. In addition, the average length of non-surgical treatment was 8 months to resolve foot pain. Therefore, clinicians need to make a clinical decision with regard to the timing of surgical treatment after counseling symptomatic patients with AN. Concerning the imaging modality, plain radiography is not sufficient to understand the pathology of symptomatic patients with AN. Based on the patient’s symptoms and history, additional imaging studies, including CT and MRI should be considered to identify the origin of foot pain.

In the present case, we did not remove the small ossicle itself and osteotomized the prominent part of the navicular tuberosity with the advancement of the TPT. In the present case, preoperative examinations did not detect the presence of tibialis posterior tendinitis. The tenderness to the prominent navicular bone was obvious without pain along the course of TPT Additionally, the preoperative radiographic findings did not show specific findings of the pes planus (Meary’s angle, 19.8 degrees; calcaneal pitch angle, 4.6 degrees). It remains under debate whether or not the pes planus would affect the clinical outcomes following the surgical treatment of symptomatic ANs. Sun et al. recently reported that midterm surgical outcomes were not different between patients with and without flatfoot after fusion for painful type 2 ANs [17]. On the contrary, Gan et al. reported that subtalar arthrodesis without the Kidner procedure relieved foot pain for pediatric flexible flatfoot combined with symptomatic type 2 AN [18]. Although the postoperative follow-up duration was not long, the present patient was free from pain at the latest follow-up examination. Jasiewicz et al. reported the surgical outcomes of the simple excision of the ossicle for five patients with type 1 AN [7]. The authors found that among the three types of AN, the postoperative pain reduction after simple excision was smallest in the type 1 group and speculated that the poor outcomes could be attributed to the need to discontinue TPT during surgery. Although the details of CT or MRI findings were not reported in this study, the study findings may suggest that simple excision of the ossicle in patients with type 1 AN may not be effective at completely resolving a patient’s foot pain. To the best of our knowledge, no other previous studies have reported on surgical outcomes for patients with type 1 AN. We cannot draw a definitive conclusion regarding the need for excision of the ossicle in patients with type 1 AN as this was a single case and we did not report on the outcomes of control patients who underwent excision of the ossicle concomitant with osteotomy of the prominent navicular and advancement of the TPT. Further studies are needed to evaluate whether the simple excision of the ossicle for patients with type 1 AN can be a radical surgical treatment.

## 4. Conclusions

We present a case of Veitch type 1 AN in which osteotomy of the prominent navicular tubercle with advancement of the TPT, without excision of the ossicle, was performed with favorable short-term outcomes.

## Data Availability

The data presented in the present study are available from the corresponding author upon request.
